# Mosquitocidal effect of ivermectin-treated nettings and sprayed walls on *Anopheles gambiae s.s.*

**DOI:** 10.1038/s41598-024-63389-x

**Published:** 2024-06-01

**Authors:** Majidah Hamid-Adiamoh, Abdul Khalie Muhammad, Benoit Sessinou Assogba, Harouna Massire Soumare, Lamin Jadama, Moussa Diallo, Umberto D’Alessandro, Mamadou Ousmane Ndiath, Annette Erhart, Alfred Amambua-Ngwa

**Affiliations:** Medical Research Council Unit The Gambia at the London School of Hygiene & Tropical Medicine, Banjul, Gambia

**Keywords:** Malaria, Drug development

## Abstract

Ivermectin (IVM) has been proposed as a new tool for malaria control as it is toxic on vectors feeding on treated humans or cattle. Nevertheless, IVM may have a direct mosquitocidal effect when applied on bed nets or sprayed walls. The potential for IVM application as a new insecticide for long-lasting insecticidal nets (LLINs) and indoor residual spraying (IRS) was tested in this proof-of-concept study in a laboratory and semi-field environment. Laboratory-reared, insecticide-susceptible Kisumu *Anopheles gambiae* were exposed to IVM on impregnated netting materials and sprayed plastered- and mud walls using cone bioassays. The results showed a direct mosquitocidal effect of IVM on this mosquito strain as all mosquitoes died by 24 h after exposure to IVM. The effect was slower on the IVM-sprayed walls compared to the treated nettings. Further work to evaluate possibility of IVM as a new insecticide formulation in LLINs and IRS will be required.

## Introduction

Malaria remains a public health problem with an estimated 249 million cases globally in 2022^1^. The large-scale deployment of the current insecticide-based vector control strategy has remarkably contributed to decreasing the malaria burden^1^. Indeed, between 2000 and 2015, it is estimated that long-lasting insecticidal nets (LLINs) and indoor residual spraying (IRS) together accounted for up to 78% of the observed reduction in malaria cases^2,3^, although insecticide resistance may revert such gains. Currently, malaria vector control relies mainly on six insecticide classes, namely: pyrethroids, organochlorines, organophosphates, carbamates, neonicotinoids and pyrroles^2^, each having a different mode of action. Pyrethroids target the mosquitoes’ voltage-gated sodium (VGS) channels^4^; while organochlorines target both the VGS and gamma-aminobutyric acid (GABA)-gated channels^4^. The organophosphates and carbamates primarily target the mosquitoes’ synaptic acetylcholinesterases (AChE)^5^, whereas the target of the neonicotinoids is nicotinic acetylcholine receptor (nAChR)^6^ and pyrroles disrupt mosquitoes’ respiratory pathways^7^. Due to the large and sustained use of these insecticides, resistance has been amplified by selection pressure; and is now widespread among all vector species^8^. Hence, alternative insecticides with novel modes of action are needed for vector control.

Recently, ivermectin (IVM), an endectocide drug used in single dose mass drug administration for the control of onchocerciasis and lymphatic filariasis, has been proposed as a potential tool for malaria vector control due to its ability to reduce the survival of mosquitoes and inhibit egg and parasite development when ingested orally by mosquitoes feeding on treated humans or cattle^9,10^. Indeed, the mosquitocidal effect of IVM on malaria vectors-when administered orally to humans, has been investigated both at individual and community levels. In Kenya, a randomized control trial showed reduced survival of mosquitoes fed on blood of adults treated with IVM combined with dihydroartemisinin-piperaquine (DP), an artemisinin-based combination therapy (ACT)^11^ . In The Gambia, mass drug administration (MDA) of IVM and DP, reduced by 80% the incidence of clinical malaria and by 60% the prevalence of malaria infection as determined by molecular methods^12^. Mosquito mortality at 7, 14 and 21 days post-treatment was significantly higher for those fed on blood samples from individuals treated with IVM compared to the control group, with the highest mosquito mortality observed at day 7. Likewise, the lethal effect of IVM on mosquitoes fed on cattle treated with IVM has been shown in several studies^9,13–16^.

IVM, when ingested with blood meal, targets the mosquitoes’ glutamate-gated chloride ion channel (GluCl) in the muscle and nerve cells to exert neuronal signals that manifest in mosquitoes’ paralysis and death^17^. IVM is attractive as it could circumvent insecticide resistance in the most efficient malaria vector, *Anopheles gambiae*, because the GluCl target site is different from that of the current chemical insecticides, the VGS and GABA-gated channels, AChE and nAChR^18^.

Most studies on the mosquitocidal effect of IVM have mainly focused on its effects when administered orally to humans and animals. There is limited knowledge on the effect of direct exposure of vectors to IVM through proven vector control approaches, *i.e.,* on bed nets (as LLINs) and wall surfaces (as IRS). Given its highly lipophilic nature, IVM can easily transverse vector cell membranes to deliver a lethal effect^19^. Direct delivery of IVM through bed nets and sprayed walls in combination with pyrethroids would be a feasible and innovative vector control approach that would counteract the current rise of resistance to pyrethroids^20^. This proof-of-concept study assessed the mosquitocidal effect of IVM-treated nettings and walls on *An. gambiae*-the main malaria vector in sub-Saharan Africa.

## Materials and methods

### Mosquito line

*An. gambiae* Kisumu strain routinely maintained in the insectaries at the medical research council unit the Gambia (MRCG) at London school of hygiene and tropical medicine was used for all experiments. The mosquito strain was originally sourced from the centre national de recherche et de formation sur le paludisme (CNRFP) in Burkina Faso and has been adapted at the MRCG insectary since 2019. The colony is maintained by blood feeding 5–7 day old adults with sheep blood using Hemotek artificial membrane feeding system (Hemotek Limited, United Kingdom) and reared in the insectary which is maintained at standard conditions (temperature: 27 ± 2 °C, humidity: 70 ± 10% and photoperiod L:D: 12:12 h). This strain has never been exposed to any insecticide and is considered a susceptible control strain for insecticide susceptibility testing with regular quality control assessment for phenotypic resistance and molecular species. All experiments were conducted within the insectary and 3–5 days old female mosquitoes only were used to assess IVM mosquitocidal activities.

### Preparation of IVM-impregnated filter papers

Stock solutions of 50 mg/ml (5% w/v) IVM were prepared from the powder (Sigma Aldrich, Catalogue #18898; 99.9% active ingredient) diluted in a mixture of acetone and olive oil, as carrier oil. Prior to the selection of acetone + olive as diluent and carrier oil for IVM, we attempted impregnating filter paper with IVM in acetone alone but this was drying and crystallizing faster when compared with the addition of olive oil. Therefore, the study conducted all experiments with this mixture. The acetone-olive oil mixture was prepared as recommended (1.29 ml _acetone_ + 0.71 ml _olive oil_ per filter paper)^21^. Subsequently, serial dilutions of seventeen different concentrations of working solutions ranging from 0.05 to 40 mg/ml (0.05–4% w/v) were prepared from this stock using acetone-olive oil mixture as diluent. IVM-impregnated filter papers were prepared following WHO recommendation^21^. Briefly, pieces of 12 × 15 cm Whatman® N°1 filter paper, as standard for WHO insecticide susceptibility test paper, were impregnated with 2 ml of IVM solution (test paper) or acetone-olive oil mixture (negative control paper). Impregnated papers were dried at room temperature in the dark in a clean cupboard overnight until the papers were completely dry and were used for experiments either after two days or stored at 4 °C until used.

### IVM dose–response and susceptibility experiments

Baseline susceptibility of mosquitoes to IVM on impregnated paper was assessed following a modified WHO insecticide susceptibility test protocol^22^. Tests were performed at different IVM concentrations to determine the optimal lethal concentration (LC) able to kill up to 99–100% (LC_99−100_) of mosquitoes within 24 h as a dose–response assay^21^. Briefly, four replicates of 25 female mosquitoes (each aged 2–5 days) were exposed to each concentration of IVM-impregnated paper in WHO test tubes for 1 h. Two batches of 25 female mosquitoes were exposed to two negative control papers and two 0.25% pirimiphos-methyl-impregnated papers as positive controls also for 1 h. Mosquitoes were then transferred into a holding tube and provided with 10% sugar solution and the number of dead mosquitoes was recorded at 30 min, and at 1, 2, 6, 18, 24, 48 and 72 hours post-exposure.

### Preparation of IVM-treated nettings and sprayed wall surfaces

Following the dose–response experiments, an effective concentration (EC) was identified and used to treat new unused plain netting materials (white polyester, multifilament, 100 deniers) and to spray wall surfaces in subsequent experiments. We applied similar formular for calculation of a discriminatory concentration as recommended by WHO^23^, where DC was defined as the twofold minimum concentration resulting in 100% mortality after 1 h exposure to an insecticide. Hence, IVM-treated nets were prepared as recommended by WHO^21^, where 30 × 30 cm nets were dipped briefly in 2.8% IVM solution (IVM+Acetone+olive oil). The nets were drained to remove excess IVM solution, dried on plastic sheets overnight in the dark at room temperature and were later removed and stored in cupboard when completely dry. Prior to impregnation, the nets were prewashed in clean water and soap and sun-dried. A separate batch of nets treated with acetone+olive oil mixture was also prepared as negative control. Deltamethrin (DM)-treated long-lasting netting material (Tianjin Yorkool International, China), was used as positive control. The treated nettings were used for experiments two days after being treated with IVM.

IVM-sprayed walls (15 × 15 cm spots) were prepared on unpainted plastered and mud walls of experimental huts situated at the MRCG entomological field station in Walinkunda (Central River Region). Briefly, 15 × 15 cm spots were marked out of wider spots and evenly sprayed using a handheld pressure controlled sprayer (Hudson, USA). 1.2 ml of 2.8% IVM solution was sprayed on each of the spots marked as ‘test’; Actellic 300CS (Syngenta Crop Protection AG, Basel, Switzerland) on those marked as ‘positive control’ and acetone+olive oil mixture as ‘negative control’. The spraying was done following the WHO recommendations on indoor residual wall spraying^21^. The walls were left to dry and later used for experiment after two days.

### Cone bioassays on the IVM-treated nettings and sprayed wall surfaces

Cone bioassays were done to assess mosquito survival following exposure to IVM within 72 h as modified from WHOPES protocol for cone bioassays^21^. The Kisumu strains used for the dose-response assays were also exposed to IVM for cone bioassays. Batches of 5 unfed female mosquitoes aged 2–5 days old were exposed to the treated nets (311 mg ai/m^2^ (EC) for 3 min and subsequently transferred into holding cups provided with 10% sugar solution. Mosquito mortality was recorded at 30 min, 1, 2, 6, 18, 24, 48 and 72 hours post-exposure. Ten replicates per treated nets and six replicates each of negative and positive control assays were performed, each batch of exposed mosquitoes kept in separate cups. Cone bioassays were similarly performed on the IVM-sprayed walls with mosquitoes exposed for 30 min and monitored for mortality for 72 h as described above. Five assay replicates were done for treated and untreated mud walls.

### Assessment of fecundity-inhibiting activity of IVM-treated surfaces

To assess the effect of IVM on fecundity, the survival of mosquitoes for at least 2–3 days following blood feeding is vital^24^. As an initial step, the study assessed the ability of mosquitoes to oviposit viable eggs that can develop into adults following standardized tarsal contact with IVM using the cone bioassays as described above. Mosquitoes were blood-fed 12 h pre- or post-exposure to IVM-treated surfaces. A subset of mosquitoes was also fed immediately after exposure to IVM. All mosquitoes were fed with defibrinated sheep blood (TCS Biosciences Limited, Netherlands) for approximately 2 h using Hemotek artificial membrane feeding system (Hemotek Limited, United Kingdom). Following blood feeding and IVM exposure using cone bioassays, mosquitoes were maintained on 10% sugar solution and observed for survival and oviposition. The abdominal status of all dead and surviving mosquitoes was subsequently examined.

### Data analyses

Mortality was estimated as the total number of dead mosquitoes divided by total number of exposed multiplied by 100 (#dead/#exposed mosquitoes × 100)^22^. Mortality to different concentrations of IVM was calculated at 24, 48 and 72 hours post-exposure and this was plotted in a dose-response curve. The LC_99_ value at 24 h mortality was determined from this dose-response plot using log-probit regression analysis^25^ in Stata/IC 16.0 (2019 StataCorp LP). The calculated LC_99_ value was subsequently multiplied by 2 for the final value of the EC as recommended^23^. The actual concentration of IVM as an active ingredient (ai) on the treated surfaces were also calculated from the 28 mg ai/ml in olive oil (28 × 1 ml) that was impregnated on 0.09 m^2^ of each netting and 44.8 mg ai/ml in olive oil (28 × 1.2 ml) sprayed on 0.0225 m^2^ of each wall. As IVM insecticidal activity were tested on the same Kisumu strain both initially on impregnated filtered paper and later on nettings and walls, these calculated ECs were retained to describe the results of all experiments.

Data from all replicate bioassays were pooled for analyses. The Abbot’s formula^26^ was not applied to correct for mortality if mortality among negative control was less than 5% at 24 h post-exposure. As mortality was observed at 24 h in all tested mosquitoes, this time point was used as the cut-off and reference mortality point also in line with recommendations^22,23^. Kaplan Meier survival curves were generated for the IVM-treated and control groups on the treated surfaces and survival rates by 24 h were compared using log-rank tests. The mortality outcome was coded as zero (0) for censored or survival, and 1 for non-survival (death) at any time-point. A P-value of 0.05 was considered as statistically significant.

### Modeling the effect of IVM on mosquitoes’ survival

As mosquitoes were treated with IVM on different surfaces and then observed at specific time intervals after treatment, the data were interval censored. Interval censoring in this case means that the event of interest (mosquito mortality) is not directly observed but is known to fall within some time interval. Non-parametric maximum likelihood estimation (NPMLE) of the Cox proportional hazards model^27^ as implemented in Stata 17 (StataCorp. LLC. 2021) was employed to estimate the regression coefficients of the Cox model. With the Cox proportional hazards model, treatment and treated surface types were considered as possible covariates since the experiments were standardized in controlled environments. Mud walls and Actellic (positive control) treatment were used as default reference categories. The final model explored the effect of treatment (IVM) and surface types (nettings, plastered or mud walls) along with their interaction terms on mosquitoes’ survival. Non-significant covariates from the crude analyses were not considered in the adjusted model. Given the relatively lengthy survival time of the mosquitoes in the negative controls (unobservable median survival time), they were excluded from the analyses. This allowed a comparison between IVM and the positive controls (Actellic for both walls and DM for netting materials). A significance level of 0.025 was considered in the modelling to reduce the familywise type-I error rate (FWER)^28^.

## Results

### IVM discriminating dose

**Figure 1:**
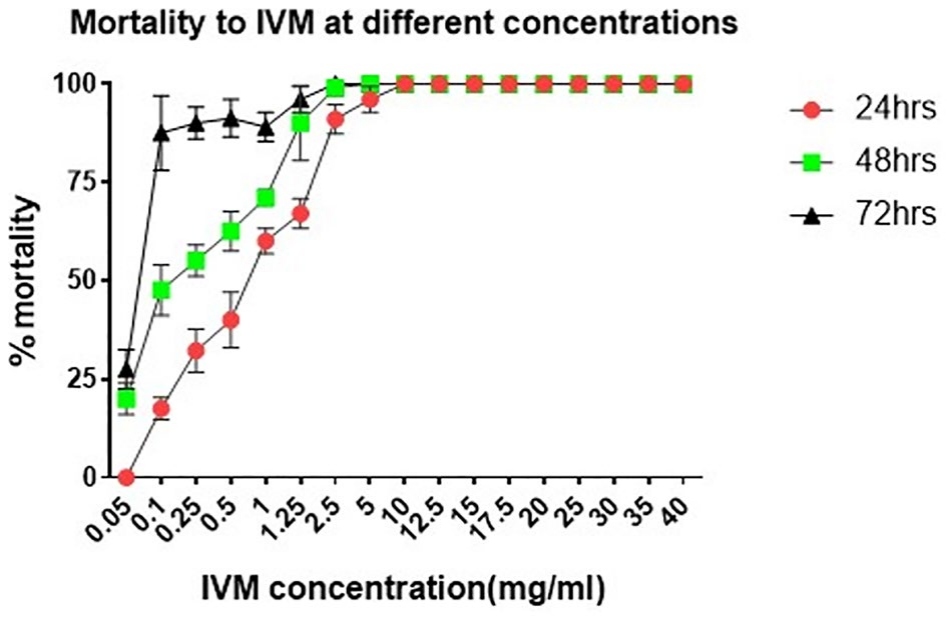
Dose-response plot showing mosquitoes’ mortality to increasing concentrations of IVM.

A total of 1,620 mosquitoes were exposed to seventeen increasing concentrations of IVM on impregnated filter papers (Supplementary Table S1). Susceptibility to IVM was dose dependent (Fig. 1), with the mortality rate from 24 h rapidly plateauing at 100% under increasing IVM concentrations between 0.05 and 5 mg/ml. Mosquitoes were fully susceptible (24 h-mortality: 100%) to IVM at concentrations ranging from 10 to 40 mg/ml (1–4% w/v) (Fig. 1). An LC_99_ value of 14 mg/ml (1.4% w/v) IVM was obtained from the log-probit regression analysis, and this was multiplied by 2 to obtain an DC of 28 mg/ml (2.8%) (95% CI 1.9–3.7%).

The detailed mortality data are presented in Supplement Table 1.

**Table 1:** Mortality of *An. gambiae* kisumu strain within 72 h. post-exposure to different IVM-treated surfaces.

(n/N) Percentage mortality (95% CI)				
Treatment surface	Post-exposure time (hour)	Test specimens	Positive control	Negative control
Nettings (N = 150)	30 min	(70/150) 46.7 (38.9–54.6)	(23/30) 76.7 (59.1–88.2)	(0/30) 0.0 (0.0–11.4)
	1	(137/150) 91.3 (85.7–94.9)	(24/30) 80.0 (62.7–90.5)	(0/30) 0.0 (0.0–11.4)
	2	(146/150) 97.3 (93.3–99.0)	(29/30) 96.7 (83.3–99.4)	(0/30) 0.0 (0.0–11.4)
	6	(150/150) 100.0 (97.5–100.0)	(30/30) 100.0 (88.6–100.0)	(0/30) 0.0 (0.0–11.4)
	18	(150/150) 100.0 (97.5–100.0)	(30/30) 100.0 (88.6–100.0)	(0/30) 0.0 (0.0–11.4)
	24	(150/150) 100.0 (97.5–100.0)	(30/30) 100.0 (88.6–100.0)	(1/30) 3.3 (0.6–16.7)
	48	(150/150) 100.0 (97.5–100.0)	(30/30) 100.0 (88.6–100.0)	(4/30) 13.3 (5.3–29.7)
	72	(150/150) 100.0 (97.5–100.0)	(30/30) 100.0 (88.6–100.0)	(5/30) 16.7 (7.3–33.6)
Plastered walls (N = 150)	30 min	(33/150) 22.0 (16.1–29.3)	(9/30) 30.0 (16.7–47.9)	(0/30) 0.0 (0.0–11.4)
	1	(63/150) 42.0 (34.4–50.0)	(19/30) 63.3 (45.5–78.1)	(0/30) 0.0 (0.0–11.4)
	2	(94/150) 62.7 (54.7–70.0)	(30/30) 100.0 (88.6–100.0)	(0/30) 0.0 (0.0–11.4)
	6	(137/150) 91.3 (85.7–94.9)	(30/30) 100.0 (88.6–100.0)	(0/30) 0.0 (0.0–11.4)
	18	(150/150) 100.0 (97.5–100.0)	(30/30) 100.0 (88.6–100.0)	(0/30) 0.0 (0.0–11.4)
	24	(150/150) 100.0 (97.5–100.0)	(30/30) 100.0 (88.6–100.0)	(0/30) 0.0 (0.0–11.4)
	48	(150/150) 100.0 (97.5–100.0)	(30/30) 100.0 (88.6–100.0)	(5/30) 16.7 (7.3–33.6)
	72	(150/150) 100.0 (97.5–100.0)	(30/30) 100.0 (88.6–100.0)	(5/30) 16.7 (7.3–33.6)
Mud walls (N = 75)	30 min	(5/75) 6.7 (2.9–14.7)	(15/30) 50.0 (33.2–66.8)	(0/30) 0.0 (0.0–11.4)
	1	(9/75) 12.0 (6.4–21.3)	(18/30) 60.0 (42.3–75.4)	(0/30) 0.0 (0.0–11.4)
	2	(19/75) 25.3 (16.9–36.2)	(30/30) 100.0 (88.6–100.0)	(0/30) 0.0 (0.0–11.4)
	6	(40/75) 53.3 (42.2–64.2)	(30/30) 100.0 (88.6–100.0)	(0/30) 0.0 (0.0–11.4)
	18	(75/75) 100.0 (95.1–100.0)	(30/30) 100.0 (88.6–100.0)	(0/30) 0.0 (0.0–11.4)
	24	(75/75) 100.0 (95.1–100.0)	(30/30) 100.0 (88.6–100.0)	(0/30) 0.0 (0.0–11.4)
	48	(75/75) 100.0 (95.1–100.0)	(30/30) 100.0 (88.6–100.0)	(6/30) 20.0 (9.5–37.3)
	72	(75/75) 100.0 (95.1–100.0)	(30/30) 100.0 (88.6–100.0)	(8/30) 26.7 (14.2–44.4)

### Reduced survival of mosquitoes exposed to IVM-treated nettings and sprayed walls

**Figure 2:**
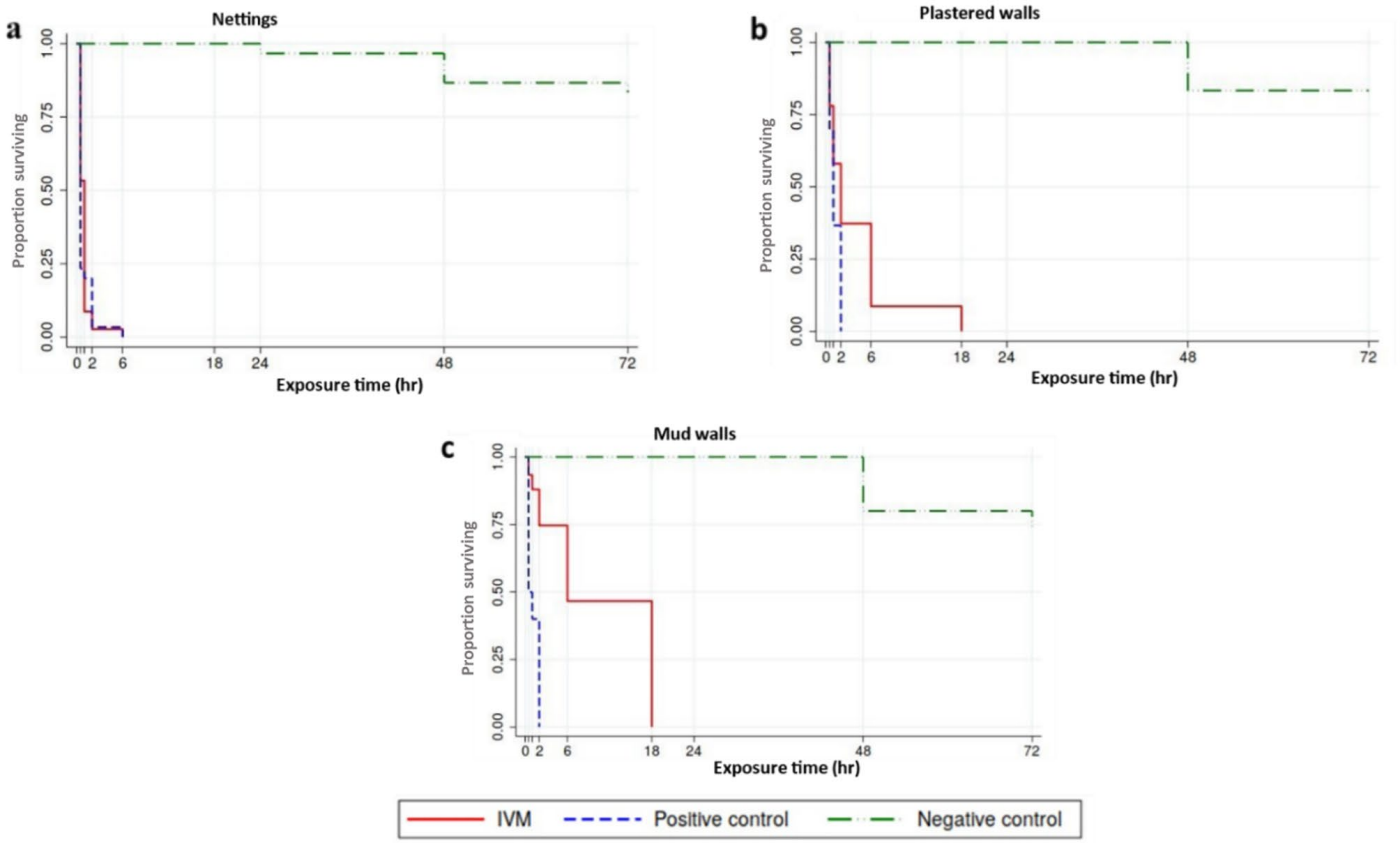
Kaplan Meier survival curves of mosquitoes on IVM-treated surfaces.

At the EC of 2.8% w/v (95% CI 1.9–3.7%) (Nettings: 311.11 mg ai/m^2^ ,Walls: 1,493 mg ai/m^2^), IVM had a significant effect on mosquito survival with a median survival time of 1 h (IQR: 30 min, 6 h). No mosquitoes survived (mortality: 100%) IVM-treated nettings by 6 h post-exposure (Table 1; Fig. 2a). Mosquito survival on the IVM-treated nettings was similar to that of DM-treated nettings (positive control) (100% mortality at 6 h). Only 3.3% (95%CI 0.1–17.2%) mosquitoes exposed to untreated nettings (negative control) died by 24 h and the observed differences in mosquito survival between treated (IVM or positive control) and untreated nettings were statistically significant (Log rank *X*^2^ = 34.54, p < 0.001).

IVM-induced mortality was also observed for mosquitoes exposed to sprayed plastered and mud walls. On the sprayed plastered walls, all mosquitoes exposed to IVM died by 18 h (Fig. 2b) while all those exposed to positive control died within 2 h of exposure. The same was observed on mud walls (Fig. 2c). Therefore, mosquitoes survived significantly longer when exposed to IVM-sprayed walls (plastered or mud) compared to positive control sprayed walls (Log rank *X*^2^ = 32.71, p < 0.001). Overall, mosquito survival on the IVM-sprayed mud walls (Fig. 2C) was slower (compared to the sprayed plastered wall), although 100% mortality was achieved on both walls within 18 h.

### IVM-treated netting associated with highest mosquitoes’ mortality (Cox model)

Overall, in the pooled analysis, all mosquitoes exposed to IVM died before the last observation time point of 24 h. Within the first 30 min, 155 (27.9%) mosquitoes died, resulting in left-censored observations; with the remaining 400 (72.1%) being interval censored. The univariate Cox regression model identified treatment (IVM/positive control) and surface type as significantly associated with mosquito mortality (Table 2). IVM showed a hazard ratio of 0.47 (95% CI 0.35–0.63) when compared with the positive control group while nettings had a hazard ratio of 6.64 (95% CI 4.91–8.96), and plastered walls of 2.15 (95% CI 1.62–2.84), when compared with mud walls.

**Table 2:** Mortality hazard of IVM *versus* controls by treatment group and surface type (Proportional Cox regression model).

Variable	Category	Crude HR (95% CI)	p value	Adjusted HR (95% CI)*	p value
Treatment	DM/actellic	1		1	
	IVM	0.47 (0.35–0.63)	< 0.001	0.11 (0.06–0.19)	< 0.001
Surface type	Mud walls	1		1	
	Nettings	6.64 (4.91–8.96)	< 0.001	1.56 (0.93–2.64)	0.094
	Plastered walls	2.15 (1.62–2.84)	< 0.001	0.9 (0.44–1.84)	0.779
Interaction term Treatment*Surface^§^	Actellic-Mud walls			1	
	IVM-Nettings			8.27 (4.20,-16.28)	< 0.001
	IVM-Plastered walls			3.69 (1.66–8.19)	0.001
Effect of treated surface type within the IVM treatment group	IVM-Nettings			12.92 (7.69–18.14)	< 0.001
	IVM-Plastered			3.33 (2.12–4.55)	< 0.001
	IVM-Mud walls			1	
Effect of IVM vs positive control treatment on each surface type	IVM-Nettings°			0.89 (0.64–1.23)	0.47
	IVM-Plastered°			0.40 (0.22–0.72)	0.002
	IVM-Mud walls°			0.11 (0.06–0.19)	< 0.001

^§^Asignificant interaction term was found between treatment and surface type, therefore the effect of IVM treatment versus positive control is computed for each surface type. °The baseline category is the same for surface treated with positive control.

In the multivariable Cox regression model, we identified surface type and treatment along with the interaction between them as the final model. Indeed, significant interaction terms were detected between IVM-treated nettings [HR = 8.27 (95% CI 4.20–16.28); p < 0.001], and with IVM-sprayed plastered walls [HR = 3.69 (95% CI 1.66–8.19); p = 0.001], both compared to Actellic-sprayed mud walls. Furthermore, IVM had a similar effect on mosquito mortality compared to DM when applied on nettings [HR = 0.89 (95% CI 0.64–1.23); p = 0.47], while this was not the case on plastered and mud walls, where the hazard with IVM was respectively, 60% lower [HR = 0.40 (95% CI 0.22–0.72); p = 0.002] and 89% lower [HR = 0.11 (95% CI 0.06–0.19); p < 0.001], relative to the positive control. Alternatively, against the IVM-treated surfaces, the mosquitocidal effect of IVM-treated nettings was significantly higher [HR = 12.92 (95% CI: 7.69–18.14); p < 0.001] than the effect of IVM-sprayed plastered walls [HR = 3.33 (95% CI 2.12–4.55); p < 0.001], both relative to IVM-sprayed mud walls. This was not the case in the group of positive control-treated surfaces where the mosquitocidal effect of treated nettings [HR = 1.56 (95% CI 0.93–2.64); p = 0.094] or sprayed plastered walls [HR = 0.90 (95% CI 0.44–1.84); p = 0.779] was not significantly different from the effect on treated mud walls.

### IVM inhibited mosquito blood feeding

**Figure 3:**
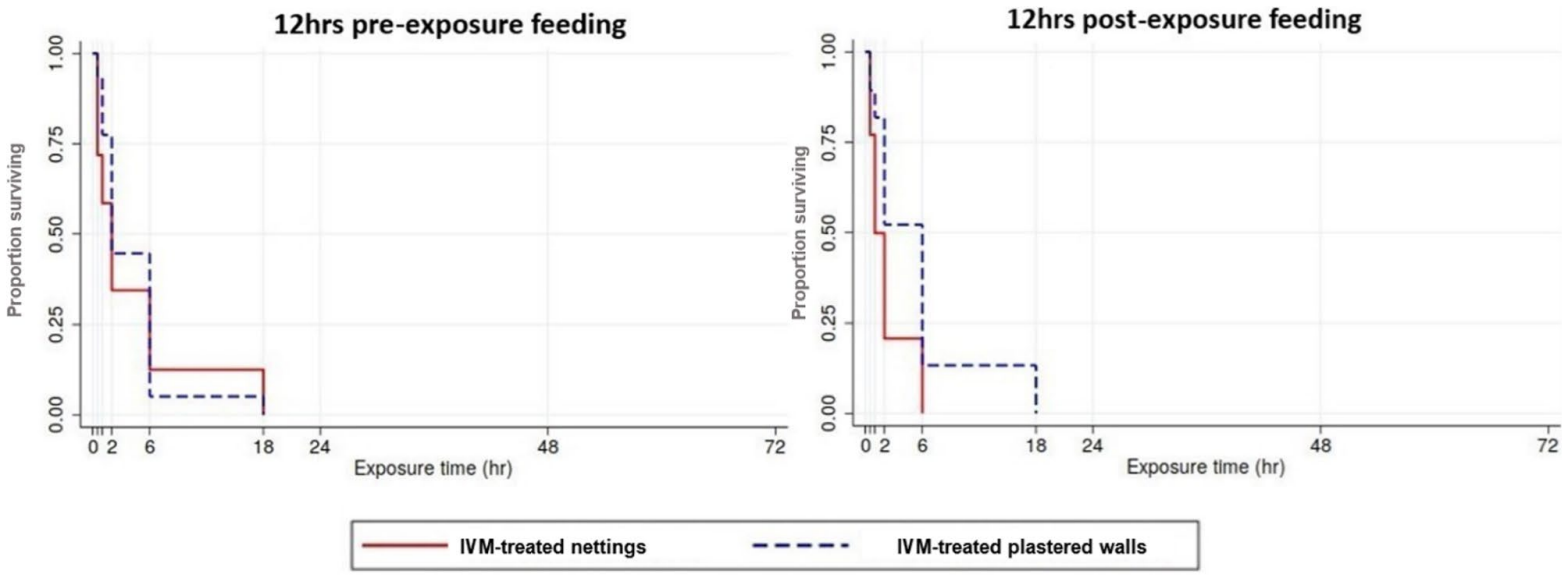
K-M survival curves of mosquitoes blood-fed 12 h pre-and post-IVM exposure.

None of the IVM-exposed mosquitoes survived beyond 24 h, regardless of the exposure being pre- or post-feeding (Fig. 3). A similar response was observed in the DM-exposed mosquitoes, whereas the majority (mortality: 3–10%) of untreated mosquitoes survived until 24 h. The survival of the mosquitoes fed 12 h post-IVM exposure on nettings was relatively shorter (2 h) compared to those fed pre-IVM exposure (18 h) (Log rank *X*^2^ = 64.79, p < 0.001). The pattern was similar on plastered walls but with longer survival time (6 h) compared to nettings. Overall, fecundity could not be measured due to the short survival of the mosquitoes exposed to IVM.

The abdominal status of 50 mosquitoes which were blood-fed immediately post-IVM exposure was examined. None of those exposed to IVM-treated nettings was able to feed, while 16% (95% CI 7.2–29.1%) of those exposed to IVM-sprayed plastered walls could feed and become fully engorged (Table 3). On the other hand, for the positive controls, 12% (95% CI 4.5–24.3%) of those exposed to DM-treated nettings and 50% (95% CI 35.5–64.5%) of those exposed to Actellic-sprayed plastered walls were fully engorged. Most (> 90%) of the negative control mosquitoes exposed on both surfaces were fully engorged.

**Table 3:** Mosquitoes’ feeding status immediately after exposure to IVM-treated nettings and plastered (unpainted) walls.

Treatment surface	Group	Total unfed mosquitoes	Total fed mosquitoes	Proportion (%) of fed mosquitoes (95% CI)
		(N = 50)	(N = 50)	(N = 50)
Nettings	IVM	50	0	0 (0.0.07)
	Negative control	3	47	94.0 (83.5–98.7)
	Positive control	44	6	12.0 (4.5–24.3)
Plastered walls	IVM	42	8	16.0 (7.2–29.1)
	Negative control	4	46	92 .0 (80.8–97.8)
	Positive control	25	25	50.0 (35.5–64.5)

## Discussion

To the best of our knowledge, this is an early report on the direct mosquitocidal effect of IVM on *Anopheles* mosquitoes when applied on nettings or wall surfaces. Our results show that IVM on nettings and wall surfaces at the EC of 2.8% w/v was highly lethal to the mosquitoes, with none of the mosquitoes surviving beyond 24 h after exposure. Moreover, tarsal exposure to IVM inhibited mosquito blood feeding which impacted egg maturation and oviposition within 72 h timeframe required for fecundity^24^.

In our study, the effect of IVM on treated surfaces seems much stronger and faster in killing mosquitoes within 24 h than when ingested with the blood meal on treated individuals, which was shown to be an average of 7 days from previous studies^11,14,29, 30^. This is probably due to the IVM concentration that may be much lower in the blood ingested by the mosquito than on the directly treated surfaces. Indeed, the highly-lipophilic nature of IVM may allow easy cell membrane permeability in mosquito cuticle, leading to paralysis and death within a short period of time^19^. A previous study^31^ which exposed the same mosquito strain (Kisumu) to IVM using CDC bottle assay^32^ also reported 100% mortality within 24 h at lower concentrations (0.01–1.5% w/v). Therefore, our results support the use of IVM for LLINs and IRS.

We observed mortality was faster on IVM-treated nettings than that on the IVM-sprayed plastered and mud walls. The final Cox model also estimated a 13-fold higher hazard on IVM- treated nettings, while it was only threefold higher on IVM-sprayed plastered walls compared to IVM-sprayed mud walls. This confirms a differential effect of IVM treatment according to surface type. Variation in mosquito mortality on different wall surfaces has been documented from previous studies^33–35^, where mortality recorded on mud walls was consistently lower compared to plastered walls for many insecticides. This relatively higher mosquitocidal effect on nettings suggests a better IVM uptake through the mosquito cuticle, increasing its binding potential to GluCl and thus mortality^19,36^. This implies that use of IVM on LLINs could be explored as a vector control strategy similar to standard pyrethroid-treated LLINs. Indeed, future approaches could combine IVM with pyrethroids, and this could control pyrethroid resistance given that IVM and pyrethroids have different mechanisms of actions and target different genes^18^. Moreover, the mosquitocidal effect of IVM-treated LLINs could be improved by further optimizing the EC to a higher concentration, although this will require toxicological assessments.

We observed statistically significant difference in mosquito survival on Actellic-sprayed walls compared to the IVM-sprayed walls (both plastered and mud walls). This difference would be operationally irrelevant as no mosquitoes survived both insecticides by 24 h endpoint. However, this result may be because the exposure time at 30 min for cone bioassays has been optimized for the current insecticides^21,22^, which is yet to be determined for IVM. Also, it may be because IVM was previously documented to be a slow acting insecticide^31^. Hence, future efforts may optimize its exposure time as previously applied to chlorfenapyr and clothianidin^37,38^. Additionally, it is possible that because Actellic’s long-lasting formulation has been optimized to be well retained on walls and available to mosquitoes being an approved IRS product^39^, but may not be the case for IVM until evaluated. Nevertheless, given this encouraging result, the potential for IVM as an IRS insecticide would be explored with further evaluations for effects at sub-lethal doses, long-lasting formulations and residual activity among others in our future studies.

We explored IVM-induced inhibition of mosquito blood-feeding pre-and post-exposure to evaluate the possibility that mosquitoes would survive and blood feed following contact with IVM-treated surfaces. Strikingly, feeding was strongly inhibited irrespective of feeding period, suggesting that IVM induced debilitating neuronal signals sufficiently strong to disrupt blood feeding. This was previously demonstrated in a study by Kobylinski and colleagues^40^, where re-feeding episodes were delayed in *An. gambiae s.s.* following oral IVM ingestion at different concentrations. The result also suggests that IVM could offer protection to human hosts from infectious bites since blood feeding was impacted due to its rapid lethal effect as demonstrated here. More importantly, the observed blood-feeding inhibition would be beneficial for malaria control, as mosquitoes exposed to IVM-treated surfaces may not survive long enough to feed or transmit malaria. As observed, oviposition assessment was disrupted post-IVM exposure as very few mosquitoes could feed or survive to oviposit. This demonstrates a potential for IVM-treated surfaces to prevent oviposition if blood feeding is inhibited. Future investigations on fecundity-inhibitory effect of IVM would be possible at sub-lethal doses and are strongly recommended.

The observed longer survival of mosquitoes that blood fed pre-IVM exposure indicates a possible interaction of blood feeding with IVM as previously observed with pyrethroid-treated netting on field *An. gambiae*^41^. This interaction may be protecting blood fed mosquitoes from IVM on these surfaces, suggesting possible reduced insecticidal activity on blood fed mosquitoes. Notwithstanding, we observed that the exposed mosquitoes did not survive to oviposit, indicating that IVM would be effective in controlling mosquito populations whether blood fed or not before contact with IVM.

As this is a proof-of-concept study mainly aimed to generate initial data on the potential for IVM as an insecticide with a different mode of action for LLINs and IRS, the results here should facilitate further evaluations for optimal dose, long-lasting formulations, residual activity and human safety^21^. Possibility of combining IVM with a pyrethroid could also be evaluated.

### Limitations

We acknowledge that we were unable to quantitatively determine the actual dose of IVM on the treated surfaces including impregnated filter papers, using chromatography-based methods. Also, we did not assess IVM effect at sub-lethal doses as done in previous studies on oral ingestion^9,42^. Sub-lethal doses would allow assessing mosquito blood feeding and fecundity inhibition. We recommend future studies to advance the suitability of IVM for LLINs and IRS.

## Conclusions

These initial data show that IVM could be effective as a contact insecticide with promising potential to be developed as a formulation for LLINs and IRS following further investigations of its suitability. The data further suggest that IVM could have effects on vector density, blood feeding and eventually malaria transmission, if used as LLINs and IRS. Given these potentials and with further evaluations, IVM could expand the current portfolio of insecticides used against malaria vectors.

### Supplementary Information

Supplementary Tables.

## Data Availability

All data are within the paper. No supporting Information is available.
