# Supplementary material for: Mosquitocidal effect of ivermectin-treated nettings and sprayed walls on Anopheles gambiae s.s
Source: Sci Rep. 2024 Jun 1;14:12620. doi: 10.1038/s41598-024-63389-x (PMC11144240; doi:10.1038/s41598-024-63389-x)
Supplement: Supplementary file 1 — Supplementary Tables. [file 41598_2024_63389_MOESM1_ESM.docx]

**Mosquitocidal effect of ivermectin-treated nettings and sprayed walls on *Anopheles gambiae s.s.***

Majidah Hamid-Adiamoh*, Abdul Khalie Muhammad, Benoit Sessinou Assogba, Harouna Massire Soumare, Lamin Jadama, Moussa Diallo, Umberto D’Alessandro, Mamadou Ousmane Ndiath, Annette Erhart and Alfred Amambua-Ngwa

**Supplement Table S1: *Anopheles gambiae* Kisumu strain’s mortality after 1** **hour** **exposure to increasing concentrations of ivermectin (IVM) on impregnated filter papers.**

|  |  |  |  |  |
| --- | --- | --- | --- | --- |
| **IVM** | **# Mosquitoes** | **Percentage mortality (95% CI)** | | |
| **Concentration** | **Exposed** |  |  |  |
| (mg/ml) | (N) | **24 h.** | **48 h.** | **72 h.** |
| 40 | 100 | 100 | 100 | 100 |
| 35 | 100 | 100 | 100 | 100 |
| 30 | 100 | 100 | 100 | 100 |
| 25 | 100 | 100 | 100 | 100 |
| 20 | 100 | 100 | 100 | 100 |
| 17.5 | 100 | 100 | 100 | 100 |
| 15 | 100 | 100 | 100 | 100 |
| 12.5 | 100 | 100 | 100 | 100 |
| 10 | 100 | 100 | 100 | 100 |
| 5 | 100 | 96 (90-99) | 100 | 100 |
| 2.5 | 100 | 91 (84-95) | 99 (94->99) | 100 |
| 1.25 | 100 | 68 (58-76) | 90 (82-95) | 96 (90-99) |
| 1 | 100 | 60 (50-69) | 71 (61-79) | 89 (81-94) |
| 0.5 | 80 | 40 (31-50) | 63 (53-72) | 91 (84-95) |
| 0.25 | 80 | 32 (24-42) | 55 (45-64) | 90 (82-95) |
| 0.1 | 80 | 18 (12-27) | 47 (38-57) | 88 (80-93) |
| 0.05 | 80 | 0 | 20 (13-29) | 27 (19-36) |
|  |  |  |  |  |

Dose-dependent mortality of mosquitoes exposed to fourteen IVM concentrations (0.5-4% w/v) on impregnated filter paper. Overall mortality was taken at 24hr, 48hr and 72hr post-exposure and the data was used to plot dose-response curve to determine diagnostic dose. Mortality presented with 95% confidence interval.
